# Physical Activity Disparities between Native Hawaiian and Pacific Islanders and Other Racial-Ethnic Groups in the United States

**DOI:** 10.21203/rs.3.rs-8622548/v1

**Published:** 2026-06-12

**Authors:** Ming Wen, Neng Wan, Pui Yin Cheung

**Affiliations:** University of Hong Kong; University of Utah; University of Hong Kong

## Abstract

This study investigates disparities in aerobic and muscle-strengthening physical activity (PA) among Native Hawaiian and Pacific Islander (NHPI) individuals in comparison to non-Hispanic White (NHW) and other racial and ethnic groups, with particular attention to multiracial identities, gender, socioeconomic status (SES), and geographic region. Using pooled data from the 2015–2019 Behavioral Risk Factor Surveillance System (BRFSS), we applied generalized structural equation modeling to elucidate underlying behavioral patterns and mechanisms. Our findings indicate that, after adjusting for SES, NHPI men exhibit a significant advantage in meeting aerobic PA guidelines relative to NHW men. Conversely, NHPI women are significantly less likely to adhere to aerobic PA recommendations, with SES mediating a substantial portion of this disparity. Regarding muscle-strengthening PA, NHPI men demonstrated significantly higher odds of meeting guidelines compared to their NHW counterparts, a disparity that became more pronounced following SES adjustment. These results suggest that the role of SES is complex and non-uniform, functioning as a mediator or suppressor that differentially influences various subgroups. Notably, no significant racial or ethnic disparities were observed among women in muscle-strengthening activities. Regional analyses further highlighted substantial variation across the Eastern United States, West Coast, Hawaii, and Guam, underscoring the importance of geographic context. Collectively, these findings underscore that PA disparities are intricately shaped by the intersectionality of racial identity, gender, SES, and geographic location. They highlight the necessity for developing targeted, culturally tailored public health interventions that address specific barriers and leverage community strengths within these diverse populations.

## Introduction

Physical activity (PA) is a critical determinant of health, yet significant differences exist across racial and ethnic groups. Research indicates that Native Hawaiian and Pacific Islander (NHPI) individuals exhibit lower levels of PA compared to other populations, contributing to higher rates of obesity, diabetes, and cardiovascular diseases [1, 2] and lower life expectancy [3]. These differences are influenced by a complex interplay of socioeconomic, cultural, and environmental factors, including limited access to recreational facilities, cultural norms that may not prioritize exercise, and systemic barriers to health equity [4, 5]. Despite the growing recognition of these disparities, NHPIs remain underrepresented in public health research, underscoring the need for more NHPI-based evidence to inform targeted interventions and culturally responsive strategies to promote PA participation and improve health outcomes in this population. Addressing these disparities is essential to achieving health equity and reducing the burden of chronic diseases among NHPIs.

In health disparity research by race and ethnicity, published studies have primarily focused on monoracial populations, often aggregating multiracial individuals into monoracial categories, which obscures their unique experiences and health outcomes [6]. At the same time, multiracial individuals represent one of the fastest-growing demographic groups in many countries, particularly in the United States [7]. In the 1970s, approximately 1 percent of children were multiracial [8]; by 2020, that number has grown to more than 10 percent according to the U.S. Census Bureau, up from 6.9% in 2010 [9]. These individuals navigate multiple cultural, social, and racial identities, which can influence their access to healthcare, health behaviors, and experiences of discrimination [10–12]. These intersecting identities may create unique stressors or protective factors that are not captured when studying monoracial groups [13]. For instance, systemic discrimination and the internal struggle to reconcile one's identity can precipitate disproportionately high rates of mental health disorders and the adoption of unhealthy coping mechanisms [11, 14, 15]. Including multiracial communities in health equity research is vital for acknowledging diverse racial backgrounds, addressing their specific health needs, and ensuring equitable healthcare access and results [7].

In this study, our primary objective was to examine PA differences between NHPI individuals and non-Hispanic White (NHW) and other racial and ethnic groups, with particular attention paid to multiracial categories. Given the well-documented gender differences in health in general [16, 17] and in PA in specific [18, 19], we also explored gender-specific disparity patterns in PA. Additionally, we investigated the extent to which socioeconomic status (SES), as indicated by household income and educational attainment, explained the PA disparities. While extensive research has addressed the potential mediating role of SES in racial and ethnic health disparities [20, 21], limited work has focused on exploring this issue within the NHPI population.

Finally, we assessed whether PA disparities vary across regions, including the Eastern United States, the West Coast, Hawaii, and Guam. We distinguished Hawaii and Guam from the continental states because NHPI individuals in Hawaii and Guam often have unique cultural and demographic characteristics compared to their counterparts living in continental US states. Hawaii and Guam are places where NHPI populations are a majority or have high concentrations, which may influence their PA patterns differently than NHPI minorities in the mainland US [22].

## Methods

### Data source

This study used pooled data from the 2015, 2017, and 2019 Behavioral Risk Factor Surveillance System (BRFSS), focusing on respondents aged 25 or older to obtain a sufficiently large and representative sample of working age adults, in particular NHPI. Established in 1984 by the Centers for Disease Control and Prevention (CDC), the BRFSS is an annual statewide telephone and cellular surveillance survey and the world’s largest continuously conducted health surveillance system [23]. We excluded the 2023 BRFSS data because it no longer included the “Which one of these groups would you say best represents your race? ” variable, which is essential for applying the Bratter and Gorman (2011) classification method in our analysis. BRFSS didn’t probe PA questions in even number of years and 2021 (due to COVID). Thus, BRFSS 2015, 2017, and 2019 are the most recent waves of BRFSS including the preferred race that best describes you and PA variables with large enough aggregate sample for NHPI, especially multiracial NHPI. Since this is an observational study using only deidentified participant data, the University of XXX Human Research Ethics Committee has confirmed that no ethical approval is required.

### Measures

#### PA Outcomes

The study assessed two PA outcomes: adherence to aerobic PA guidelines and adherence to muscle-strengthening PA guidelines. Adherence to aerobic PA was defined as engaging in at least 150 minutes of moderate-intensity aerobic exercise (or at least 75 minutes of vigorous-intensity aerobic exercise), in accordance with the recommendations outlined in the 2008 Physical Activity Guidelines for Americans [24]. Respondents were grouped into three categories: no exercise, inadequate exercise (less than the recommended amount), and meeting or exceeding the recommended amount. Based on the same guidelines, adherence to the muscle-strengthening exercise recommendation was defined as engaging in activities of moderate or greater intensity that target all major muscle groups on 2 or more days per week. Response options were grouped in two categories: inadequate muscle-strengthening exercise and adequate muscle-strengthening exercise.

#### Race and ethnicity

The primary racial and ethnic group of focus is NHPI. Relying solely on monoracial racial and ethnic categories may mask important health disparities [25]. To mitigate this limitation, we leveraged multiracial status data from the BRFSS to expand our classification into five distinct groups: (1) monoracial non-Hispanic white, (2) multiracial non-Hispanic white, (3) monoracial NHPI, (4) multiracial NHPI, and (5) other racial and ethnic groups. Respondents could choose to select one or more racial groups (“Which one or more of the following would you say is your race? Mark all that apply.”) Those who selected more than one racial group could choose a preferred race (“Which one of these groups would you say best represents your race?”) and were designated multiracial [race]. For instance, a respondent who selected both white and NHPI with a preferred race of NHPI would be coded as multiracial NHPI in the analysis.

#### Other independent variables

The analysis included household income and educational attainment as mediating factors. Education levels were categorized as under high school, high school or equivalent, some college, and college or above. Household income was divided into the following eight (8) brackets: under $10,000, $10,000-$14,999, $15,000-$19,999, $20,000-$24,999, $25,000-$34,999, $35,000-$49,999, $50,000-$74,999, and $75,000 or above. The state identifier corresponded to one of the 50 states, Washington DC, or Guam. Puerto Rico was excluded from the analysis as it did not have a significant NHPI population. Age and survey year were included in the analysis as control variables. Gender-specific modeling was performed.

### Statistical analysis

Path analytic models using generalized structural equation modeling (GSEM) with survey weight adjustment were conducted to examine our research questions [26]. The two PA outcomes were predicted by age and racial and ethnic groups in four gender-specific equations: (1) age, racial and ethnic groups and state identifier as covariates (Model 1); (2) education added to Eq. (1) (Model 2); (3) household income added to Eq. (1) (Model 3); and (4) education and income added to Eq. (1) (Model 4). Indirect effects were estimated using the difference in coefficients method [27]. Since the PA outcomes were either ordinal or binary, all equations were estimated using ordinal logistic or logistic regressions. Path model results were reported separately for the male and female subsamples.

We addressed missing data using multivariate imputation by chained equations (MICE) implemented via the mice package in R [28]. Prior to imputation, we listwise deleted observations (n = 284) with a missing value for gender, our stratification variable, to prevent inconsistencies in this key categorical variable across imputed datasets. The imputation model used a random forest algorithm, applied separately by year, in place of standard linear regression. This approach was selected for its flexibility in modeling complex interactions and nonlinear relationships across diverse variable types, which reduces bias and enhances the efficiency of the estimates [29, 30]. We generated and pooled 10 imputed datasets for subsequent analysis, according to the procedure outlined by von Hippel [31]. All estimations were done using survey weights provided by the BRFSS.

To examine the third research question regarding the moderating role of region on PA disparities, we classified participants' regions of residence into four groups: Eastern US, West Coast, Hawaii, and Guam. The Eastern US includes the Census regions of Northeast, Midwest, and South. The West Coast encompasses the Mountain and Pacific regions, excluding Hawaii. Hawaii and Guam were analyzed separately, as Native Hawaiian and Pacific Islander (NHPI) populations are predominantly concentrated in these areas. We estimated four separate models for each region, stratified by gender, using GSEM to compare PA patterns among NHPI across regions. Covariates included age and survey year.

## Results

Table 1 displays the weighted sample statistics of 2015, 2017 and 2019 BRFSS respondents aged 25 or older (N = 1,219,246), stratified by gender. A lower percentage of NHPI women (46.8%) and a higher percentage of NHPI men (54.0%) met the aerobic PA recommendation, compared to non-NHPI women (49.2%) and non-NHPI men (51.5%). The prevalence rates of adherence to the muscle-strengthening PA recommendation were generally lower, with higher percentage of NHPI adherence than non-NHPI adherence (28.3% for NHPI women vs. 26.7% for non-NHPI women and 41.0% for NHPI men vs. 33.3% for non-NHPI men). The sample consisted predominantly of single-race non-Hispanic whites (NHW; 65.7% for both men and women). Among NHPI participants, who constitute about 0.4% of the total sample, approximately 16.5% of NHPI men and 20.3% of NHPI women identify as multiracial. NHPI individuals are generally younger than their non-NHPI counterparts and tend to have lower levels of education and income. Geographically, most participants in each group reside in the Eastern US, with about 23–25% living on the West Coast. Notably, NHPI participants are more likely to live in Hawaii and Guam than non-NHPIs.

Table 2 displays the GSEM results predicting adherence to aerobic PA guidelines among men. The base model (Model 1a), which did not include socioeconomic covariates, showed no significant differences in adherence between NHPI and NHW men, regardless of monoracial or multiracial status, nor between monoracial and multiracial NHPI men. However, respondents categorized in the "Other" racial group—primarily comprising Black, Asian, and Hispanic/Latino individuals—exhibited significantly lower odds of meeting aerobic PA recommendations compared to monoracial NHPI men (OR = 0.77, *p* < .01). After controlling for SES, including educational attainment and household income, in Model 4a, both monoracial and multiracial NHW men demonstrated significantly lower odds of adherence relative to monoracial NHPI men (OR = 0.80, *p* < .05). Still no significant difference was observed between monoracial and multiracial NHPI men. These findings suggest that socioeconomic factors may suppress the association, obscuring an underlying advantage in aerobic PA adherence among NHPI men compared to NHW men.

In contrast to patterns observed among men, women who identified as monoracial NHW (OR = 1.63, *p* < 0.001) and multiracial NHW (OR = 1.57, *p* < 0.001) were all significantly more likely to meet the aerobic PA recommendation than monoracial NHPI women in Model 1a. These associations were largely mediated by SES factors as indicated by the attenuation of odds ratios from Model 1a to Model 4a. The mediating effect was substantial among monoracial and multiracial NHW groups (with odds ratios reduced by 62% and 44%, respectively). Conversely, no statistically significant disparities were revealed when comparing monoracial NHPI women with either multiracial NHPI women or those categorized as "Other."

Table 3 presents the GSEM results predicting adherence to aerobic PA recommendations by region, using the Model 1 configuration (not including education and household income). Among men, while no significant racial/ethnic differences were found in the Eastern U.S., several significant associations were present elsewhere. On the West Coast, men in the 'Other' category had significantly lower odds of adherence than monoracial NHPI men (OR = 0.54, *p* < .01). Monoracial NHW men in Hawaii (OR = 1.45, *p* < .01) and multiracial NHW men in Guam demonstrated markedly higher odds of adherence (OR = 3.45, *p* < .01).

For women, the significant disparities observed in Table 1 appeared primarily driven by the patterns exhibited in the Eastern U.S., Hawaii, and Guam, with no significant associations found on the West Coast. Notably, the disparity between multiracial NHPI and monoracial NHPI women appeared in Guam only, with the former exhibiting higher odds of adherence.

Table 4 presents the results on adherence to muscle-strengthening PA recommendations. Among men, monoracial NHWs (OR = 0.79, *p* < 0.05) and men in the Other group (OR = 0.79, *p* < 0.05) exhibited lower odds of adherence compared to monoracial or multiracial NHPIs, based on Model 1b. After adjusting for education and household income, the disparity for monoracial NHW men became more pronounced (OR = 0.63, p < 0.001 in Model 4b), indicating that socioeconomic resources may suppress the disadvantages observed among NHPI men. Among women, no significant racial or ethnic differences in adherence were detected.

Table 5 presents adherence to muscle-strengthening PA recommendations across four regions. Among men, compared to monoracial NHPIs, men in the Other group on the West Coast had lower odds of adherence (OR = 0.63, *p* < 0.05), similar to what we saw for adherence to aerobic PA recommendations. At the same time, monoracial NHW men in Guam were significantly more likely to meet muscle-strengthening PA guidelines than their monoracial NHPI counterparts (OR = 1.72, *p* < 0.01). For women, compared to monoracial NHPIs, significantly higher adherence was observed among multiracial NHPIs in the Eastern U.S. (OR = 3.93, *p* < 0.05), monoracial NHWs in Hawaii (OR = 1.59, *p* < 0.001) and Guam (OR = 2.21, *p* < 0.001), as well as multiracial NHWs in Hawaii (OR = 1.47, *p* < 0.05).

## Discussion

This study provides novel insights into aerobic and muscle-strengthening PA disparities comparing NHPI populations with other racial-ethnic groups, with a particular focus on monoracial versus multiracial identities, gender differences, socioeconomic factors, and regional variations. By leveraging the 2015, 2017, and 2019 BRFSS data, this analysis addresses critical gaps in the literature concerning NHPI populations and multiracial individuals, who are often underrepresented in health disparity research ^1,7^. The findings highlight complex patterns that underscore the importance of culturally and contextually tailored public health strategies to address PA disparities within these communities.

The most notable finding is the contrasting pattern of aerobic PA by gender. Initial models revealed no significant difference between NHWs and NHPIs among men. However, after adjusting for SES, NHPI men exhibited a significant advantage over their NHW counterparts. This indicates a suppression effect, where the generally lower SES among NHPI men conceals their higher underlying propensity for engaging in aerobic PA. These results challenge the common deficit model often applied to minority health, suggesting instead that cultural strengths or environmental factors within NHPI communities may promote aerobic activity among men, independent of economic resources.

In stark contrast, women displayed a more traditional disparity pattern. NHPI women had significantly lower odds of meeting aerobic guidelines compared to all other female groups in the baseline model. This highlights that the NHPI experience is not monolithic and that gender roles, cultural expectations, and access barriers likely influence men and women differently. The near-complete attenuation of these disparities after adjusting for SES factors suggests that socioeconomic inequities are a primary driver of lower aerobic activity participation among NHPI women. Additionally, for both women and men, multiracial versus monoracial identity did not significantly influence the observed disparities in aerobic PA.

For adherence to muscle strengthening recommendation, the advantage of NHW men manifested in the monoracial NHWs rather than multiracial NHWs. The persistence and apparent widening of the disparity among monoracial NHW men following SES adjustment suggest that factors beyond socioeconomic access—such as cultural, social, or behavioral determinants—may contribute to the NHPI men’s greater engagement with muscle-strengthening activities than monoracial NHW men. This could reflect cultural differences in the types of activities pursued (e.g., traditional NHPI activities that build strength versus gym-based weightlifting) or differences in occupational physical demands. The absence of significant disparities among women for this outcome further emphasizes that the drivers of muscle-strengthening behavior are distinct from those for aerobic activity and may be less influenced by racial and SES factors for women.

The regional analyses provide an important contextual layer, underscoring that place and local culture are fundamental to understanding PA patterns. The observation that the pronounced disparities in aerobic PA among women in the full sample were largely absent in the West Coast subsample suggests that the experiences of NHPI women in this region differ markedly from those in other areas of the US. It can be speculated that factors such as greater cultural integration and community support, increased acculturation and exposure to Western health norms, and improved access to parks and walkable neighborhoods likely contribute to this pattern. These environmental and social advantages may create more opportunities and incentives for aerobic activity among NHPI women on the West Coast, mitigating disparities seen elsewhere.

It is not surprising that NHW men are particularly advantaged in aerobic PA in Hawaii and Guam, and in muscle-strengthening PA in Guam, while these disparities were less pronounced in the continental US. This pattern supports our expectation that the unique cultural environment of Hawaii and Guam influences PA engagement in ways not observed in mainland settings. In these regions, NHPI communities often preserve strong cultural traditions and social networks that may not place the same emphasis on or prioritize certain types of PA, such as organized sports or gym-based exercises. These cultural norms can shape health behaviors differently than in the mainland US, contributing to the regional differences in PA participation among NHPI men.

Notably, multiracial NHPI individuals exhibited PA patterns that were largely congruent with those of their monoracial counterparts. A significant deviation, however, was observed among women in the Eastern US; in this region, multiracial NHPI women demonstrated an approximate four-fold likelihood of meeting strength-training guidelines compared to monoracial NHPI, NHW, and other racial subgroups. This distinct regional variation underscores the necessity of investigating the socioeconomic, cultural, and environmental determinants specific to this demographic. Elucidating the drivers behind this pronounced engagement in muscle-strengthening, particularly relative to aerobic activity, may yield critical insights for designing targeted health promotion strategies

This study has several limitations. First, the cross-sectional design of the BRFSS precludes any causal inferences. Second, PA is self-reported and subject to recall and social desirability biases. Third, while we advanced racial classification by including multiracial groups, the "Other" category remains heterogeneous, aggregating diverse populations such as Black, Asian, and Latino individuals. Future research should disaggregate these groups. Furthermore, our SES measures, though standard, are limited. Future studies would benefit from more granular measures of wealth, occupational status, and neighborhood-level SES and PA-related environmental features. Finally, while we controlled for region, we lacked specific data on cultural practices (e.g., participation in traditional activities) or perceived discrimination, which are likely key mediators and moderators of the relationships we observed.

In conclusion, our findings transcend a simplistic narrative of health disparities, revealing a complex interplay of race, multiracial identity, gender, socioeconomic status, and geography. We demonstrate that socioeconomic factors suppress aerobic PA advantages among NHPI men, while contributing to disadvantages among NHPI women. Furthermore, our results underscore that geographic region acts as a notable moderator of PA disparity patterns, highlighting the pivotal role of the local environment in shaping health behaviors. Additionally, we find that multiracial identity can influence disparities across race, gender, and region, particularly in muscle-strengthening activities, emphasizing the nuanced ways in which identity intersects with other social determinants to impact physical activity engagement.

These insights have important implications for public health practice and policy. Interventions designed to increase PA within NHPI communities must be tailored and specifically targeted to address the unique needs of these populations. Promoting aerobic activity among NHPI women necessitates policies that confront underlying socioeconomic barriers to participation. Conversely, strategies for NHPI men could focus on leveraging existing cultural strengths and encouraging engagement in activities they already practice. Our study underscores the importance of continued research on multiracial populations, as neglecting this group risks obscuring critical heterogeneity and perpetuating health disparities among one of the fastest-growing demographic segments. Ultimately, public health strategies should be context-specific and developed in collaboration with local communities to ensure cultural relevance and effectiveness.

## Figures and Tables

**Table 1.** Sample Descriptive Statistics based on 10 Multiply Imputed Datasets (N=1,219,246)

	Non-NHPI women			NHPI women			Non-NHPI men			NHPI men		
	%	Mean	SD	%	Mean	SD	%	Mean	SD	%	Mean	SD
Adhering to aerobic exercise recommendation												
*No exercise*	31.2			35.5			29.3			28.9		
*Under 150 minutes aerobic exercises*	19.5			17.7			19.2			17.1		
*At least 150 minutes aerobic exercises*	49.2			46.8			51.5			54.0		
Adhering to muscle-strengthening exercise recommendation												
*No exercise*	73.3			71.7			66.7			59.0		
*At least 2 days of muscle-strengthening exercises per week*	26.7			28.3			33.3			41.0		
Race/ethnicity												
*Non-Hispanic white (monoracial)*	65.9			0			66.0			0		
*Non-Hispanic white (multiracial)*	0.5			0			0.6			0		
*NHPI (monoracial)*	0			79.7			0			83.5		
*NHPI (multiracial)*	0			20.3			0			16.5		
*Other racial/ethnic groups*	33.6			0			33.4			0		
Age		51.93	16.14		45.79	14.52		50.37	15.72		42.29	12.91
Education												
*Below high school*	13.2			16.8			14.4			22.0		
*High school*	25.6			33.7			27.5			33.8		
*Some college*	31.3			28.3			28.5			24.8		
*College or above*	29.9			21.3			29.6			19.4		
Household income												
*Below $10,000*	6.5			11.2			4.4			6.0		
*$10,000–$14,999*	5.9			6.5			4.2			5.5		
*$15,000–$19,999*	8.4			11.2			6.5			10.1		
*$20,000–$24,999*	9.8			10.3			8.2			13.2		
*$25,000–$34,999*	10.9			10.1			9.6			10.5		
*$35,000–$49,999*	12.9			11.7			13.5			12.3		
*$50,000–$74,999*	14.4			14.5			15.6			12.4		
*$75,000 or above*	31.3			24.4			38.0			29.9		
Region												
*Eastern US*	76.6			48.0			75.8			53.6		
*West coast*	23.0			24.4			23.7			24.7		
*Hawaii*	0.4			21.3			0.4			16.2		
*Guam*	0.02			6.4			0.02			5.4		

**Table 2.** GSEM predicting meeting aerobic exercise recommendation using ordinal logistic regression

	Model 1a (no mediator)	Model 2a (with education)	Model 3a (with income)	Model 4a (with both mediators)
**Among men**				
Race/ethnicity				
*Non-Hispanic white (monoracia)*^1^	1.08	0.87	0.87	**0.80** *
	[0.89,1.32]	[0.71,1.06]	[0.72,1.07]	[0.66,0.98]
*Non-Hispanic white (multiracial))*^1^	0.97	0.83	0.86	**0.80** *
	[0.77,1.21]	[0.66,1.04]	[0.68,1.07]	[0.64,1.00]
*NHPI (multiracial)* ^1^	1.04	0.98	0.98	0.96
	[0.76,1.44]	[0.72,1.33]	[0.71,1.34]	[0.70,1.30]
*Other racial/ethnic groups*^1^	**0.77** **	**0.73** **	**0.75** **	**0.73** **
	[0.63,0.94]	[0.60,0.89]	[0.61,0.92]	[0.60,0.89]
No. of observations	466,719	466,719	466,719	466,719
**Among women**				
Race/ethnicity				
*Non-Hispanic white (monoracial)*^1^	**1.63** ***	**1.34** **	**1.32** *	**1.24** *
	[1.33,1.99]	[1.10,1.64]	[1.07,1.62]	[1.01,1.52]
*Non-Hispanic white (multiracial)*^1^	**1.57** ***	**1.36** **	**1.41** **	**1.32** *
	[1.25,1.97]	[1.08,1.70]	[1.11,1.78]	[1.04,1.66]
*NHPI (multiracial)* ^1^	1.19	1.19	1.16	1.17
	[0.86,1.65]	[0.85,1.65]	[0.84,1.62]	[0.84,1.63]
*Other racial/ethnic groups*^1^	1.05	1.01	1.04	1.02
	[0.86,1.28]	[0.83,1.23]	[0.84,1.28]	[0.83,1.25]
No. of observations	608,859	608,859	608,859	608,859

Note: 95% confidence interval in bracket. Odds ratios shown. Bold coefficients indicate significant differences from Equation 1a estimates. Estimates are based on 10 multiply imputed datasets.

1 Reference category: NHPI (monoracial).

Covariates include year, age, education, household income, and state identifiers.

* *p* < 0.05

** *p* < 0.01

*** *p* < 0.001

**Table 3.** GSEM by region predicting meeting aerobic exercise recommendation using ordinal logistic regression

	Model 1a (Eastern US)	Model 1a (West coast)	Model 1a (Hawaii)	Model 1a (Guam)
**Among men**				
Race/ethnicity				
*Non-Hispanic white (monoracia)*^1^	1.16	0.82	**1.45** **	1.26
	[0.89,1.52]	[0.57,1.18]	[1.11,1.89]	[0.91,1.73]
*Non-Hispanic white (multiracial)*^1^	0.95	0.84	0.85	**3.45** **
	[0.71,1.28]	[0.55,1.28]	[0.61,1.19]	[1.35,8.82]
*NHPI (multiracial)* ^1^	1.87	0.52	1.15	0.70
	[0.81,4.30]	[0.23,1.19]	[0.85,1.56]	[0.43,1.13]
*Other racial/ethnic groups*^1^	0.86	**0.54** **	0.89	0.78
	[0.66,1.13]	[0.38,0.79]	[0.69,1.14]	[0.59,1.3]
No. of observations	351,165	104,489	8,980	2,085
**Among women**				
Race/ethnicity				
*Non-Hispanic white (monoracial)*^1^	**1.79** ***	1.25	**1.71** ***	**2.07** ***
	[1.33,2.40]	[0.86,1.82]	[1.34,2.17]	[1.37,3.11]
*Non-Hispanic white (multiracial)*^1^	**1.74** ***	1.16	1.26	1.75
	[1.26,2.41]	[0.76,1.77]	[0.92,1.72]	[0.85,3.62]
*NHPI (multiracial)* ^1^	2.29	0.93	0.97	**1.52** *
	[0.81,6.42]	[0.33,2.66]	[0.72,1.28]	[1.01,2.29]
*Other racial/ethnic groups*^1^	1.17	0.82	0.93	1.14
	[0.87,1.57]	[0.56,1.20]	[0.74,1.17]	[0.88,1.48]
No. of observations	470,064	126,294	10,104	2,397

Note: 95% confidence interval in bracket. Odds ratios shown. Bold coefficients indicate significant differences from Model 1a (Hawaii) estimates. Estimates are based on 10 multiply imputed datasets.

1 Reference category: NHPI (monoracial).

Covariates include year and age.

* *p* < 0.05

** *p* < 0.01

*** *p* < 0.001

**Table 4.** GSEM predicting meeting muscle strengthening recommendation using ordinal logistic regression

	Model 1b (no mediators)	Model 2b (with education)	Model 3b (with income)	Model 4b (with both mediators)
**Among men**				
Race/ethnicity				
*Non-Hispanic white (monoracia)*^1^	**0.79** *	**0.67** ***	**0.68** ***	**0.63** ***
	[0.64,0.97]	[0.54,0.81]	[0.55,0.84]	[0.52,0.78]
*Non-Hispanic white (multiracial))*^1^	0.91	0.81	0.84	0.79
	[0.72,1.15]	[0.64,1.02]	[0.67,1.07]	[0.63,1.00]
*NHPI (multiracial)* ^1^	1.08	1.05	1.04	1.03
	[0.76,1.52]	[0.74,1.47]	[0.74,1.48]	[0.73,1.46]
*Other racial/ethnic groups*^1^	**0.79** *	**0.75** **	**0.77** *	**0.75** **
	[0.64,0.96]	[0.62,0.92]	[0.63,0.95]	[0.62,0.93]
No. of observations	479,032	479,032	479,032	479,032
**Among women**				
Race/ethnicity				
*Non-Hispanic white (monoracial)*^1^	1.10	0.92	0.96	0.89
	[0.87,1.40]	[0.72,1.17]	[0.75,1.21]	[0.70,1.13]
*Non-Hispanic white (multiracial)*^1^	1.21	1.05	1.12	1.04
	[0.93,1.58]	[0.80,1.38]	[0.86,1.47]	[0.79,1.36]
*NHPI (multiracial)* ^1^	1.22	1.23	1.22	1.23
	[0.81,1.85]	[0.82,1.86]	[0.81,1.83]	[0.82,1.85]
*Other racial/ethnic groups*^1^	0.88	0.85	0.89	0.85
	[0.70,1.12]	[0.66,1.08]	[0.70,1.13]	[0.67,1.09]
No. of observations	626,432	626,432	626,432	626,432

Note: 95% confidence interval in bracket. Odds ratios shown. Bold coefficients indicate significant differences from Equation 1b estimates. Estimates are based on 10 multiply imputed datasets.

1 Reference category: NHPI (monoracial).

Covariates include year, age, education, household income, and state identifiers.

* *p* < 0.05

** *p* < 0.01

*** *p* < 0.001

**Table 5.** GSEM by region predicting meeting muscle strengthening recommendation using ordinal logistic regression

	Model 1b (Eastern US)	Model 1b (West coast)	Model 1b (Hawaii)	Model 1b (Guam)
**Among men**				
Race/ethnicity				
*Non-Hispanic white (monoracia)*^1^	0.77	0.70	1.00	**1.72** **
	[0.58,1.02]	[0.48,1.01]	[0.78,1.30]	[1.18,2.49]
*Non-Hispanic white (multiracial)*^1^	0.80	0.93	0.89	1.02
	[0.59,1.10]	[0.60,1.44]	[0.62,1.26]	[0.38,2.70]
*NHPI (multiracial)* ^1^	1.09	2.04	0.93	1.27
	[0.35,3.35]	[0.79,5.28]	[0.69,1.25]	[0.79,2.05]
*Other racial/ethnic groups*^1^	0.83	**0.63** *	0.80	1.09
	[0.62,1.11]	[0.43,0.92]	[0.63,1.03]	[0.81,1.45]
No. of observations	360,786	106,982	9,141	2,123
**Among women**				
Race/ethnicity				
*Non-Hispanic white (monoracial)*^1^	1.10	0.94	**1.59** ***	**2.21** ***
	[0.79,1.54]	[0.59,1.48]	[1.22,2.08]	[1.45,3.37]
*Non-Hispanic white (multiracial)*^1^	1.20	0.99	**1.47** *	1.30
	[0.84,1.72]	[0.59,1.65]	[1.03,2.10]	[0.46,3.63]
*NHPI (multiracial)* ^1^	**3.93** *	2.08	0.88	0.89
	[1.14,13.51]	[0.81,5.31]	[0.65,1.20]	[0.56,1.41]
*Other racial/ethnic groups*^1^	0.95	0.65	1.04	1.18
	[0.68,1.33]	[0.41,1.03]	[0.80,1.35]	[0.87,1.60]
No. of observations	484,274	129,452	10,275	2,431

Note: 95% confidence interval in bracket. Odds ratios shown. Bold coefficients indicate significant differences from Model 1b (Hawaii) estimates. Estimates are based on 10 multiply imputed datasets.

1 Reference category: NHPI (monoracial).

Covariates include year and age.

* *p* < 0.05

** *p* < 0.01

*** *p* < 0.001

## Data Availability

The BRFSS data can be accessed at the U.S. Centers for Disease Control and Prevention (CDC) website. The replication package containing Stata and R script files can be accessed at: https://dataverse.harvard.edu/dataset.xhtml?persistentId=doi:10.7910/DVN/XUPRUE
